# Genetic insights into gut microbiota and risk of prostatitis: a Mendelian randomization study

**DOI:** 10.3389/fmicb.2024.1389715

**Published:** 2024-04-12

**Authors:** Pengfei Qin, Yanmei He, Huan Shao, Dawei Jiang

**Affiliations:** Department of Urology, Zhejiang Chinese Medical University Affiliated Jiaxing TCM Hospital, Jiaxing, China

**Keywords:** prostatitis, gut microbiota, Mendelian randomization, causal relationship, genome-wide association study

## Abstract

**Background:**

The dysbiosis of gut microbiota (GM) is considered a contributing factor to prostatitis, yet the causality remains incompletely understood.

**Methods:**

The genome-wide association study (GWAS) data for GM and prostatitis were sourced from MiBioGen and FinnGen R10, respectively. In the two-sample Mendelian randomization (MR) analysis, inverse variance weighting (IVW), MR-Egger, weighted median, simple mode, weighted mode, and maximum likelihood (ML) methods were utilized to investigate the causal relationship between GM and prostatitis. A series of sensitivity analysis were conducted to confirm the robustness of the main results obtained from the MR analysis.

**Results:**

According to the IVW results, genus *Sutterella* (OR: 1.37, 95% CI: 1.09–1.71, *p* = 0.006) and genus *Holdemania* (OR: 1.21, 95% CI: 1.02–1.43, *p* = 0.028) were associated with an increased risk of prostatitis. The phylum Verrucomicrobia (OR: 0.76, 95% CI: 0.58–0.98, *p* = 0.033) and genus *Parasutterella* (OR: 0.84, 95% CI: 0.70–1.00, *p* = 0.045) exhibited a negative association with prostatitis, indicating a potential protective effect. Sensitivity analysis showed that these results were not affected by heterogeneity and horizontal pleiotropy. Furthermore, the majority of statistical methods yielded results consistent with those of the IVW analysis.

**Conclusions:**

In this study, we identified two GM taxon that might be protective against prostatitis and two GM taxon that could increase the risk of developing prostatitis. These findings could potentially provide a valuable theoretical basis for the future development of preventive and therapeutic strategies for prostatitis.

## 1 Introduction

Prostatitis, characterized by inflammation or swelling of the prostate gland, is a disease predominantly affecting adult males of all age groups and emerges as the most common urological diagnosis among men under the age of 50 (Yebes et al., 2023). Approximately half of all men are estimated to encounter symptoms of prostatitis at some stage in their lives (Naber and Weidner, 2000). Among all prostatitis cases, more than 90%−95% are diagnosed as chronic nonbacterial prostatitis (CNP), representing 25% of all visits to urological clinics worldwide (Khan et al., 2017). Moreover, a study involving 3,000 patients aged 20–59 revealed that prostatitis patients are increasingly younger, with 78% of them under the age of 40, and the prevalence of the condition rises with age (Liang et al., 2004). Patients with chronic prostatitis (CP) often manifest symptoms such as pelvic pain, voiding dysfunction, sexual disorders, and psychosocial distress (Verze et al., 2016). The clinical management of CP commonly involves the use of antibiotics, alpha-blockers, phytotherapy, and hormonal therapy (Khan et al., 2017). Nevertheless, treating CP poses a significant challenge for urologists due to its controversial etiology. In addition, the application of these traditional medicines can increase the risk of gastrointestinal problems, allergies and kidney damage (Heta and Robo, 2018). Therefore, there is a demand for innovative therapeutic strategies beyond conventional medicines.

The intestine serves as the primary site for digestion and absorption, while also functioning as the largest immune organ (Thomson et al., 2020). The gut microbiota (GM) is a diverse group of microorganisms that reside in the human gastrointestinal tract, comprising about 40 trillion microbes representing over 1,000 microbial species. Recent studies have extensively explored the association between GM and human diseases (Illiano et al., 2020). Its crucial role in maintaining both local and systemic tissue homeostasis has garnered recognition (Gensollen et al., 2016). Moreover, the metabolic by-products and ligands produced by gut commensal bacteria possess the capacity to regulate and fine-tune the innate and adaptive immune system's development and functionality, thereby aiding in defense against infections induced by diverse pathogens (Abt et al., 2012).

The concept of the gut-prostate axis was initially introduced in 2005, indicating the close relationship between the prostate and the intestine, particularly during the treatment of prostatitis (Abascal et al., 2005). Shoskes et al. (2016a,b) reported that the GM of CP patients exhibited lower alpha diversity and higher counts of *Varibaculum*. Additionally, Konkol et al. (2019) observed an increase in Odoribacter, Clostridiaceae, and Rikenellaceae, along with a decrease in Lactobacillus, Lachnospiraceae, and Bacteroides uniformis in the GM of rats with CP.

To date, however, the existence of a causal relationship between GM and prostatitis remains uncertain due to potential confounding factors and the possibility of reverse causation. Mendelian randomization (MR) presents as an emerging genetic epidemiological approach that uses summary data from genome-wide association study (GWAS) as instrumental variables to statistically deduce causality from an exposure to an outcome (Smith and Ebrahim, 2003). The MR approach shares a conceptual similarity with randomized controlled trials, differing only in that patients are allocated based on their DNA genotypes. In addition, the MR approach can overcome the methodological constraints of conventional observational studies, rendering the results more robust and reliable (Emdin et al., 2017). As the number of large-scale GWASs increases rapidly and more data is available, the statistical power of MR analysis is improving significantly (Kurilshikov et al., 2021; Verma et al., 2024).

In this study, GM taxa were selected as the exposure and prostatitis as the outcome for MR analysis, aiming to investigate the causal relationship and lay a theoretical basis for further exploration of the mechanisms underlying prostatitis. Furthermore, understanding the role of specific GM taxa and their impact on prostatitis has the potential to advance biomarker identification and precision medicine for this disease.

## 2 Methods

### 2.1 Study design and assumptions of MR

**Figure 1:**
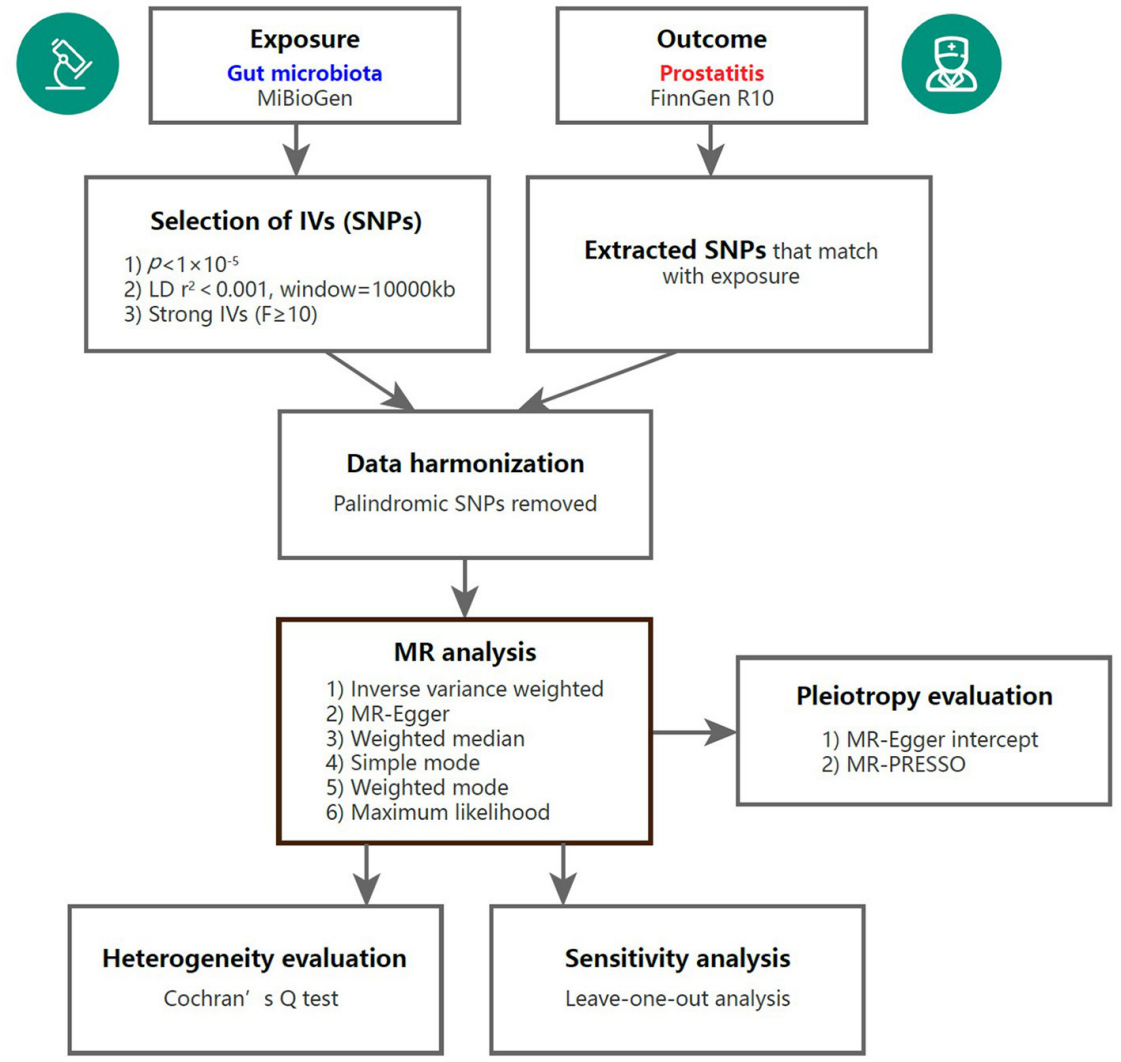
Overview of the MR analysis process. IV, instrumental variable; SNP, single nucleotide polymorphism; MR, Mendelian randomization; MR-PRESSO, Mendelian randomization pleiotropy residual sum and outlier.

To assess the causal links between gut microbiota and prostatitis, a two-sample MR analysis was conducted. The flowchart of the MR analysis is shown in Figure 1. Additionally, the MR analysis met the following three assumptions in order to minimize bias and produce reliable results: (1) Correlation assumption: the selected IVs are closely related to gut microbiota taxa; (2) Independence assumption: the IVs and confounding factors were independent of each other; (3) Exclusive assumption: the included IVs affected prostatitis only through GM taxa (Davies et al., 2018).

### 2.2 Data sources and selection of IVs

#### 2.2.1 Exposure data of GM

Kurilshikov et al. (2021) evaluated the relationship between genetic variation and GM by conducting 16S rRNA gene sequencing profiles and gathering genotyping data from 18,340 individuals, based on the MiBioGen consortium (https://mibiogen.gcc.rug.nl/). All the participants of the MiBioGen consortium was of European ancestry, sourced from 25 cohorts in 11 different nations. By examining 211 taxa (phylum, class, order, family, and genus), the GWAS study obtained 122,110 variant sites, which allowed for the analysis of GM taxonomic variance among various populations. In this study, the lowest taxonomic level was genus.

A comprehensive inspection of quality was conducted out on the SNPs in order to obtain reliable Instrumental Variables (IVs) that would ensure data robustness and results accuracy. (1) Selection of IVs at a significance level of *p* < 1 × 10^−5^ was implemented to ensure a comprehensive outcome (Jia et al., 2019; Lv et al., 2021). (2) To diminish linkage disequilibrium (LD) among the SNPs, LD-clumping was performed (*r*^2^ < 0.001, window size = 10,000 kb) on all IVs. SNPs that did not adhere to the assumption were removed. (3) The *F*-statistic for each bacterial taxa was calculated to assess the strength of the Ivs (*F* = β^2^/se^2^) (Liu et al., 2023). To minimize weak instrument bias, IVs with *F* statistic <10 were excluded (Burgess et al., 2011).

#### 2.2.2 Outcome data of prostatitis

The GWAS summary statistics for prostatitis were sourced from FinnGen Release10 (https://r10.finngen.fi/). Samples gathered by the Finnish Biological Bank's National Network were used in the FinnGen study. Genomic data was integrated with prostatitis data, comprising 4,160 cases and 130,139 controls, involving a total of 21,311,942 SNPs. The study's participants were all of European ancestry. Prostatitis diagnosis was based on the International Classification of Diseases (ICD) codes. Specifically, in ICD-10, it is denoted as N41, and in ICD-9, as 601.

In order to avoid the impact of alleles on the association between GM and prostatitis, exposure data and outcome data were harmonized by removing palindrome SNPs.

### 2.3 Statistical analysis

All statistical analysis was performed using the “TwoSampleMR” package and the “MR-PRESSO” package in R (version 4.3.2). The “forest” package was used for drawing the forest plot. Some figures have been optimized to enhance clarity of display (Chen et al., 2022). Results with *p* < 0.05 were considered statistically significant.

#### 2.3.1 MR analysis

The association between GM and prostatitis was assessed using six statistical methods: inverse variance weighted (IVW), MR-Egger, weighted median, simple mode, weighted mode and maximum likelihood (ML). IVW was used as the main analysis due to its high statistical power (Hemani et al., 2018). MR-Egger, weighted median, simple mode and weighted mode were applied as additional methods for MR analysis (Bowden et al., 2015, 2016). Furthermore, the ML was applied due to its minimal bias, particularly in small sample sizes. To determine the size of the causal effect, odds ratio (OR) and 95% confidence intervals (CI) were calculated.

#### 2.3.2 Sensitivity analysis

Heterogeneity was assessed through Cochrane's *Q*-test and *Q*-values with a *p* < 0.05 indicated the presence of heterogeneity. The global test from the MR-PRESSO estimator and the MR-Egger intercept method were used to assess pleiotropy, with *p* < 0.05 in IVs being considered as evidence of horizontal pleiotropy. To further increase the overall robustness of our results, the leave-one-out analysis was conducted to identify any significant link influenced by a single SNP (Xiang et al., 2021).

When the IVW result was statistically significant and there was no horizontal pleiotropy or heterogeneity, the GM taxa was thought to be associated with a higher risk of prostatitis.

## 3 Results

### 3.1 Selection of IVs related to gut microbiota

After LD-clumping and palindromic SNPs removal, 2,740 SNPs were finally identified as IVs related to 211 GM taxa for prostatitis (*p* < 1 × 10^−5^). All SNPs demonstrated sufficient validity (*F* statistic ranged from 14.59 to 88.43, all *F* > 10), indicating that the estimation of causal effects in our study was unlikely to be influenced by weak IVs. The key information of selected IVs was detailed in Supplementary Table S1.

### 3.2 Results of Mendelian randomization analysis

**Figure 2:**
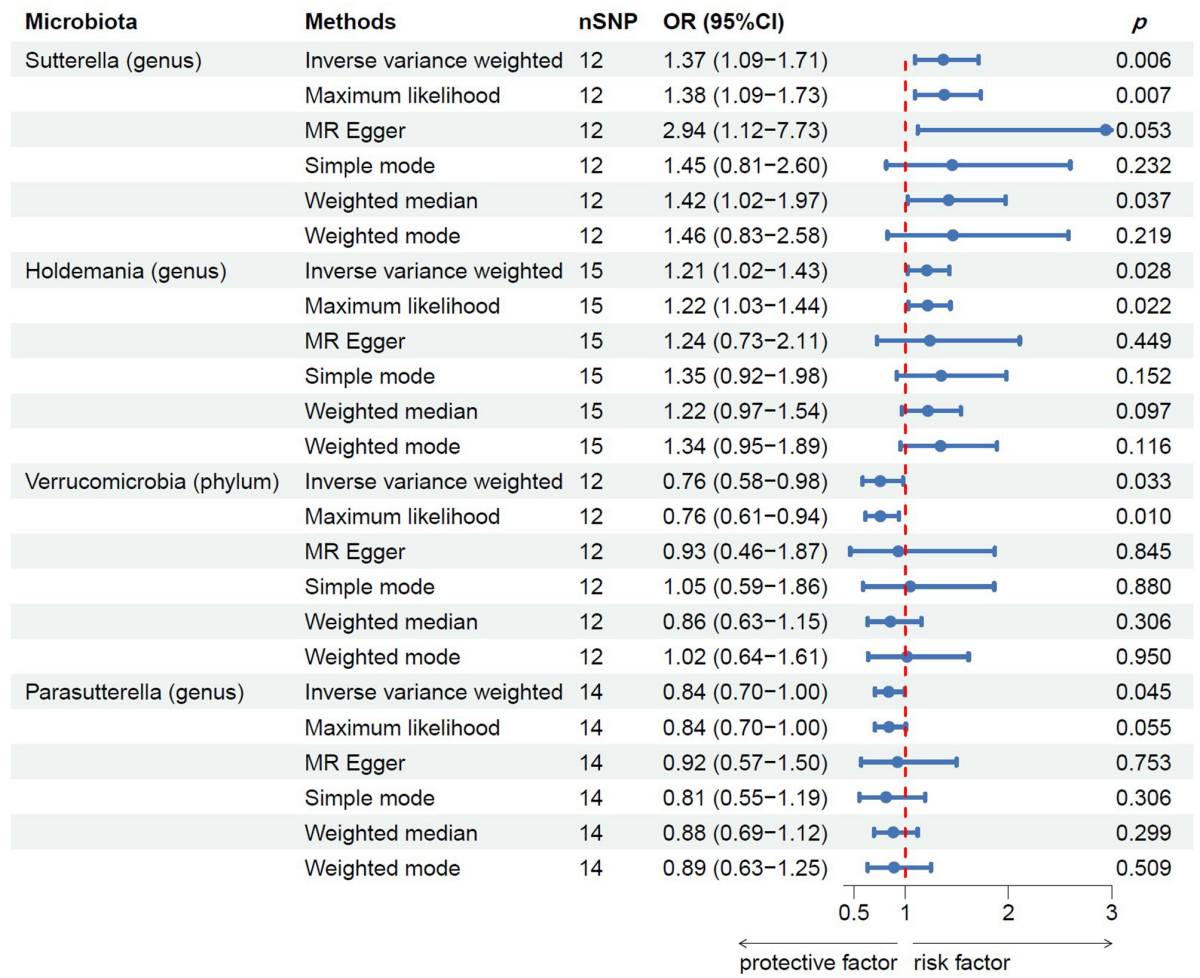
MR results of casual links between specific gut microbiota taxa and prostatitis risk. nSNP, number of single nucleotide polymorphism.

Following MR analysis, we obtained insights into the correlation between 211 GM taxa and prostatitis, with detailed data presented in Supplementary Table S2. Based on the results, we identified four GM taxa associated with the risk of prostatitis, comprising one phylum and four genera (Figure 2). At the biological phylum classification level, the IVW method results indicated that Verrucomicrobia exhibited an association with a reduced risk of prostatitis (OR: 0.76, 95% CI: 0.58–0.98, *p* = 0.033). Moving to the biological genus classification level, the MR estimates from the IVW method revealed that *Sutterella* and *Holdemania* were positively correlated with the risk of prostatitis (OR: 1.37, 95% CI: 1.09–1.71, *p* = 0.006 for *Sutterella*; OR: 1.21, 95% CI: 1.02–1.43, *p* = 0.028 for *Holdemania*), while *Parasutterella* exhibited a negative association with the risk of prostatitis (OR: 0.84, 95% CI: 0.70–1.00, *p* = 0.045). In the MR analysis of three genera, the direction and effect size of other five estimators were similar to IVW (Figure 2).

**Figure 3:**
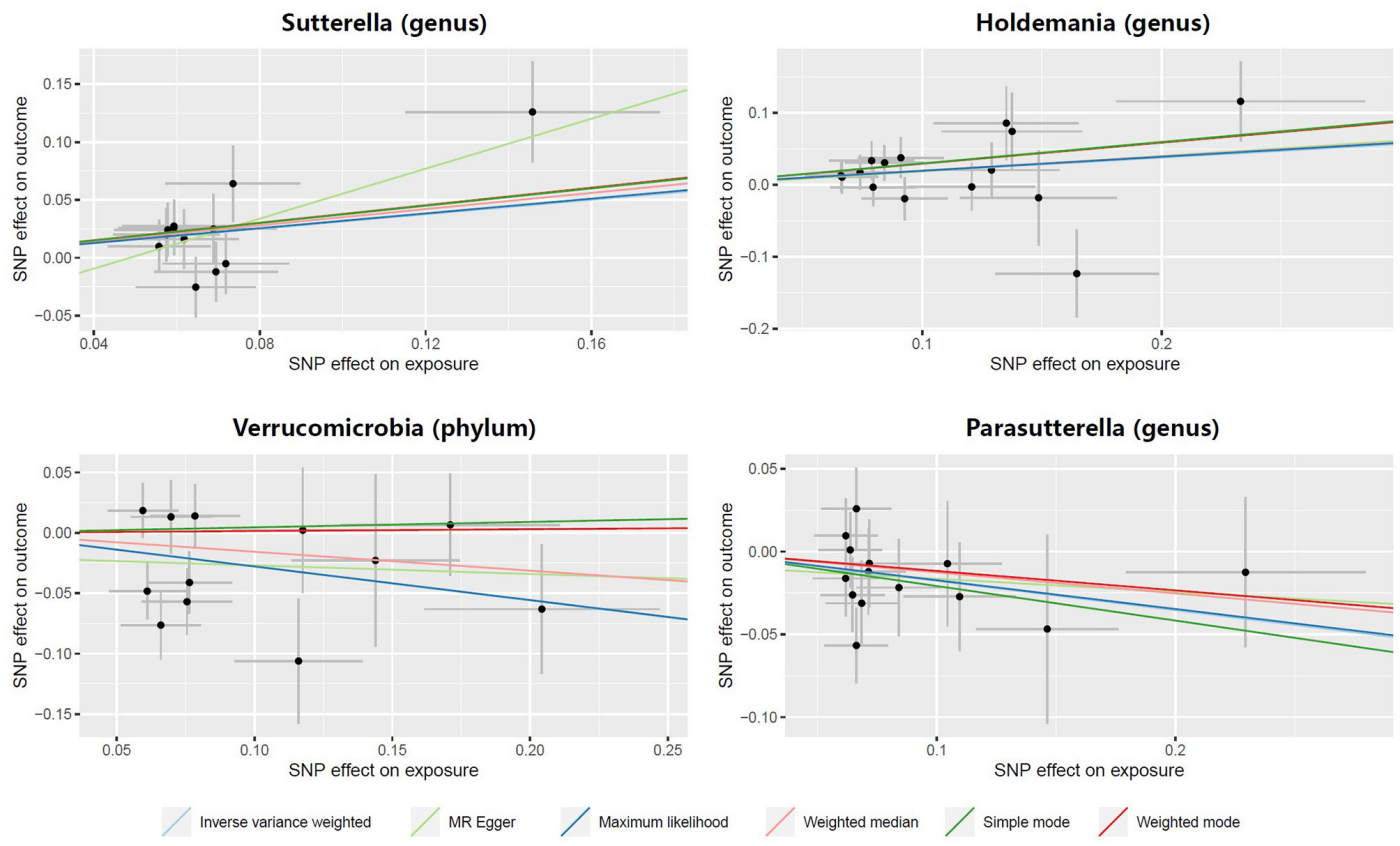
Scatter plots illustrating the causal effect of gut microbiota on prostatitis.

In both the *Sutterella* and *Holdemania* groups, scatter plots showed that an increased SNP effect was related to a higher probability of prostatitis (Figure 3). In addition, a decreased incidence of prostatitis was associated with larger SNP effects in the *Parasutterella* group. The fitted lines of the scatter plot for the Verrucomicrobia group showed inconsistent directions for several estimators. The weighted mode and simple mode showed a positive correlation, but the remaining estimators revealed a negative correlation.

### 3.3 Results of sensitivity analysis

After sensitivity analysis, the effect of accurate MR results on prostatitis in one phylum and three genera was confirmed. Egger test showed no significant horizontal pleiotropy in Verrucomicrobia (phylum, *p* = 0.539), *Sutterella* (genus, *p* = 0.141), *Holdemania* (genus, *p* = 0.927) and *Parasutterella* (genus, *p* = 0.677) for prostatitis (Table 1). Moreover, MR-PRESSO was used to further verify the statistically significant MR results to ensure the reliability of MR Egger regression. The results of global test confirmed the absence of horizontal pleiotropy in Verrucomicrobia (*p* = 0.106), *Sutterella* (*p* = 0.410), *Holdemania* (*p* = 0.427) and *Parasutterella* (*p* = 0.766) groups.

**Table 1:** Assessment of horizontal pleiotropy in the casual association.

**Microbiota**	**nSNP**	***F*-statistics**	***p*-values (Global test)**	**Intercept (Egger test)**	***p*-values (Egger test)**
*Sutterella* (genus)	12	21.05	0.410	−0.053	0.141
*Holdemania* (genus)	15	21.86	0.427	−0.002	0.927
Verrucomicrobia (phylum)	12	21.96	0.106	−0.020	0.539
*Parasutterella* (genus)	14	22.06	0.766	−0.009	0.677

**Figure 4:**
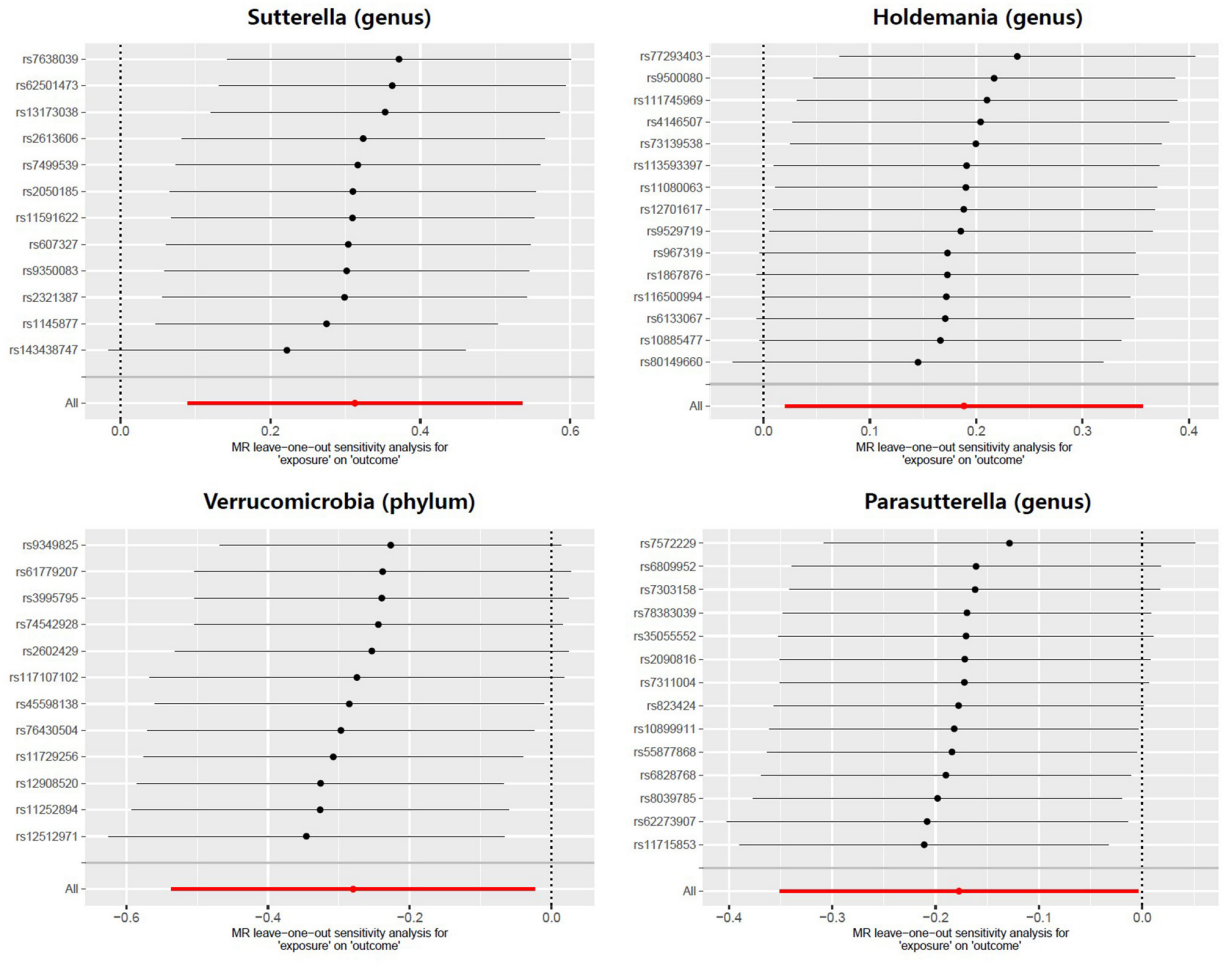
Leave-one-out analysis for the effect of individual SNPs on the correlation between the GM taxa and the risk of prostatitis. The horizontal axis represents the effect of the GM on the risk of prostatitis after excluding individual SNP. The vertical axis represents the individual SNP that have been excluded. The red line represents the overall effect of all SNPs. The dots represent the odds ratio values, the horizontal lines represent the 95% confidence intervals.

Meanwhile, there was no evidence of heterogeneity in Verrucomicrobia (IVW: *p* = 0.085; MR Egger: *p* = 0.071), *Sutterella* (IVW: *p* = 0.417; MR Egger: *p* = 0.554), *Holdemania* (IVW: *p* = 0.389; MR Egger: *p* = 0.318) and *Parasutterella* (IVW: *p* = 0.760; MR Egger: *p* = 0.704) for prostatitis (Table 2). The results of leave-one-out analysis further validated the robustness of the data (Figure 4). The IVW results were considered reliable in the absence of pleiotropy and heterogeneity. Thus, Verrucomicrobia (phylum), *Sutterella* (genus), *Holdemania* (genus), and *Parasutterella* (genus) were identified as causally related to the risk of prostatitis.

**Table 2:** Assessment of heterogeneity using Cochrane's *Q*-test.

**Microbiota**	***Q*** **-values**		***p*** **-values**	
	**IVW**	**MR Egger**	**IVW**	**MR Egger**
*Sutterella* (genus)	11.315	8.766	0.417	0.554
*Holdemania* (genus)	14.841	14.830	0.389	0.318
Verrucomicrobia (phylum)	17.843	17.149	0.085	0.071
*Parasutterella* (genus)	9.173	8.991	0.760	0.704

IVW, inverse variance weighted.

## 4 Discussion

Prostatitis is a major health problem plaguing adult men worldwide, and it can lead to a serious decline in patients' quality of life or even induce psychological disorders. Meanwhile, the etiology and pathological mechanisms of prostatitis are complex and heterogeneous (Maeda et al., 2023). Liu et al. (2022) attempted to explore the pathogenesis behind CP and conducted a multi-omics analysis using a rat model, which ultimately verified the existence of the gut-prostate axis. The GM, as the second human genome, influencing host epigenetics through multiple mechanisms, including DNA methylation programming. It has garnered significant attention in recent years for its role in the pathogenesis of various diseases (Human Microbiome Jumpstart Reference Strains Consortium et al., 2010). Researches have revealed its significant influence on the human immune system through mechanisms such as increasing immune cell populations, synthesizing short-chain fatty acids (SCFAs), enhancing oral tolerance, and modulating inflammation (Samuelson et al., 2015). Differences in GM abundance between prostatitis patients and healthy controls have been observed in numerous studies, although findings across current experiments remain inconsistent (Shoskes et al., 2016a,b; Konkol et al., 2019; Liu et al., 2021). To the best of our knowledge, this is the first MR analysis utilizing genetic data to investigate a potential causal relationship between the GM and prostatitis.

In earlier reports, higher levels of Odoribacter, Clostridiaceae, Varibaculum, Rikenellaceae, Peptococcaceae, and Allobaculum were observed in the intestines of individuals with chronic nonbacterial prostatitis as well as in rat models, with a concomitant decrease in Lactobacillus, Bacteroides, and Lachnospiraceae (Shoskes et al., 2016a,b; Konkol et al., 2019). Following this, in a multi-omics analysis conducted on a rat model of inflammatory prostate, Liu et al. found an increase in the relative abundances of Bacteroidales, Corynebacteriaceae, Corynebacterium, Prevotella, Prevotellaceae, and Rikenellaceae RC9, alongside a decrease in the relative abundance of Firmicutes, Bacilli, Coprococcus, Clostridiales, Clostridia, Turicibacteraceae, and Lachnospiracea (Liu et al., 2021, 2022). In particular, the variation in abundance of Bacteroidales and Firmicutes is significant. It is evident that there exists substantial heterogeneity in the outcomes of these studies, with some even presenting conflicting conclusions. We believe that this may stem from differences in species/ethnicity among subjects, modeling methodologies, and microbial examination techniques. This study provides insight from a genetic standpoint, indicating that increased abundance of *Sutterella* and *Holdemania* poses a risk for prostatitis, while Verrucomicrobia and *Parasutterella* might offer protective effects against the development of the condition. The research findings regarding *Sutterella* and *Parasutterella* are consistent with a recent study (Shen et al., 2024). Notably, the other two types of GM taxon have received little attention in previously reported studies on the subject.

Members of the genus *Sutterella*, as prevalent symbionts in the gastrointestinal tract, demonstrate an ability to adhere to intestinal mucosal epithelial cells. *In vitro* experiments have indicated their role in maintaining intestinal barrier function while exhibiting a mild pro-inflammatory capacity (Hiippala et al., 2016). During co-culture with intestinal epithelial cells, members of the genus *Sutterella* have been observed to induce the production of IL-8, which is considered a potential pro-inflammatory mechanism (Kaakoush, 2020). Furthermore, a theoretical viewpoint posits that *Sutterella* may compromise the effectiveness of the intestine antibacterial immune response, specifically by limiting the ability to contain intracellular bacteria, rather than directly inducing inflammation (Hansen et al., 2019). The genus *Holdemania* belongs to the phylum Firmicutes and is a relatively atypical genus. It can be found in various environments, including the gastrointestinal tract of animals. Some observational studies have also suggested a significant increase in the abundance of *Holdemania* in the intestines of patients with various inflammatory diseases such as hepatitis, neuroinflammation, and pulmonary inflammation (Zhang et al., 2023). It is speculated that changes in its abundance may affect the integrity of the intestinal barrier, facilitating the translocation of microbial products into the bloodstream, thereby triggering inflammatory responses. However, the actual role of such bacterium in the pathogenesis of prostatitis remains unexplored in research reports. The phylum Verrucomicrobia encompasses various beneficial intestinal bacteria, whose outer membrane proteins can protect interactions between cells. Members of the phylum Verrucomicrobia are believed to play a significant role in enhancing host immune responses and metabolic functions (van Niftrik and Devos, 2017). Research revealed that their metabolic products may be involved in the reduction of the inflammatory factor TNF-α and its membrane receptor TNF-R2, which may be related to the protective capacity of the phylum Verrucomicrobia against prostatitis. (Zhang et al., 2022). Regarding the genus *Parasutterella*, its bacterial metabolites haloperidol glucuronide and PPs 7-keto deoxycholic acid are considered important signaling molecules in the gut-prostate axis, promoting remission of chronic non-bacterial prostatitis in rats (Yu et al., 2022). Another study found a positive correlation between an elevation in the abundance of *Parasutterella* and serum testosterone levels (Chu et al., 2020). These microorganisms indirectly mediate the host's immune-inflammatory response by modulating hormone synthesis. Such effects have also been observed in the chronic intestinal inflammation of patients with irritable bowel syndrome (Chen et al., 2018).

While this study has shed light on the role of the gut microbiota in the pathogenesis of prostatitis, it is equally important to pay attention to the local microbial community within the urogenital tract. Wu et al. evaluated the microbiota composition in the urethral secretions and expressed prostatic secretions (EPS) of 33 male patients with CP and compared them with samples from 30 healthy individuals. The results revealed significant differences between the experimental and control groups (Wu et al., 2020). Similarly, Shoskes et al. (2016a) confirmed that in patients with CP, 17 GM taxon, including Clostridia and Bacteroidia, were over-represented, while five were under-represented. Streptococcus, Escherichia, and Pseudomonas are considered common local genera implicated in benign diseases, including chronic prostatitis. This may be related to their greater involvement in prostatic metabolism (Mjaess et al., 2023). It is noteworthy that microbial migration may also be a potential mechanism. Some researchers speculate, based on the similarity between the fecal and seminal microbiota of patients with CP, that live bacteria may migrate from the intestine to the genitourinary tract, becoming part of the local microbiota (Wang et al., 2023).

The metabolites and bioactive factors produced by GM may also act as exacerbators or suppressors of deleterious changes triggered by the local microbiome, thereby exerting an impact on prostatitis. For instance, *Sutterella* has been identified as a driving factor in the progression of diabetes, which, due to its weakening effect on immune function, may serve as a promoter of inflammation initiated by local pathogens in the prostate (Liu and Dong, 2024).

Additionally, while viruses, fungi, and archaea did not yield positive results in this study, their role as minor constituents of the gut microbiota in potentially regulating prostatic metabolism cannot be overlooked and requires further clarification. This study has several noteworthy strengths. Firstly, unlike previous research where stool samples were collected post-episode, this study establishes the temporal sequence between GM colonization and the onset of prostatitis. MR analysis is a method for exploring causality because it eliminates potential confounders and reverse causality, helping to improve the stability of our results. Secondly, the genetic variation of GM taxa utilized in the study was derived from the largest available GWAS meta-analysis, ensuring the strength and reliability of the instrumental variables selected in the MR analysis. Thirdly, any potential horizontal pleiotropy was identified and addressed through the use of MR-Egger regression intercepts and MR-PRESSO tests. Sensitivity analysis was conducted to assess the robustness of the results and provide statistical evidence of potential bias.

When interpreting the findings of this study, several limitations need to be considered in account. First of all, the causal relationships between GM and prostatitis at the species level could not be identified because the exposure dataset only included data at the genus taxonomic level. Species and subspecies within any genus may exert different or even opposite effects on host metabolism. In Wang et al.'s study on the pathogenic microbial community of prostatitis, although Gammaproteobacteria and Betaproteobacteria both belong to Proteobacteria, they exhibit opposite distribution trends and clinical significance (Wang et al., 2023). Therefore, it is necessary to conduct more comprehensive research on the impact of species within genera on prostatic metabolism. Secondly, the categorization of prostatitis subtypes was not available within the FinnGen consortium database, which limited our ability to perform subgroup analyses. The etiology, pathogens, pathogenesis, and clinical features of prostatitis vary among different types. Further subgroup analysis based on specific subtypes may yield more accurate results. Thirdly, population stratification may still cause interference even if the bulk of participants in the GWAS meta-analysis for both GM data and prostatitis data were of European ancestry. The generalization of the present findings to other ethnic groups was constrained. Future MR research on this subject should be conducted in populations outside of Europe to reconfirm the results. Finally, we were unable to perform correlation analysis of GM abundance between individuals with prostatitis and healthy controls.

## 5 Conclusions

The results of this two-sample MR study indicate that Verrucomicrobia and *Parasutterella* may be protective against prostatitis, whereas *Sutterella* and *Holdemania* may be positively associated with the risk of prostatitis. Further studies are needed to more comprehensively understand the possible beneficial or harmful effects of these GM taxa on prostatitis as well as the underlying mechanisms. Confirming the possible effect of prostatitis on GM is also important, even though there isn't much evidence to support it.

## Data availability statement

The original contributions presented in the study are included in the article/Supplementary material, further inquiries can be directed to the corresponding author.

## Ethics statement

Ethical approval was not required for the study involving humans in accordance with the local legislation and institutional requirements. Written informed consent to participate in this study was not required from the participants or the participants' legal guardians/next of kin in accordance with the national legislation and the institutional requirements.

## Author contributions

PQ: Data curation, Investigation, Writing – original draft. YH: Investigation, Project administration, Writing – review & editing. HS: Formal analysis, Software, Visualization, Writing – review & editing. DJ: Conceptualization, Methodology, Writing – review & editing.
